# Atopic dermatitis-induced failure to thrive and hypernatremic dehydration managed with dupilumab in a 5-month-old patient

**DOI:** 10.1016/j.jdcr.2024.10.042

**Published:** 2025-03-24

**Authors:** Brooke Walterscheid, Blayne Fenner, Hannah Chaudhury, Michelle Tarbox

**Affiliations:** a Department of Dermatology, Texas Tech University Health Sciences Center, Lubbock, Texas; b Texas Tech University Health Sciences Center School of Medicine, Lubbock, Texas

**Keywords:** atopic dermatitis, dupilumab, failure to thrive, hypernatremic dehydration, hypothermia, intrinsic eczema

## Introduction

Atopic dermatitis (AD) is a common chronic inflammatory skin disorder characterized by genetics, environmental factors, epidermal barrier disruption, and immune dysregulation.^1^ Rarely, severe AD induces skin barrier dysfunction resulting in protein deficiency and electrolyte abnormalities, ultimately causing failure to thrive (FTT). This case features a 5-month-old female who presented with altered consciousness due to electrolyte abnormalities, poor feeding, weight loss, and subsequent FTT. After extensive investigation, she was diagnosed with severe AD and initiated on dupilumab. This case highlights the rare sequelae of severe AD and the use of biologic therapies for infants with severe or refractory cases of AD.

## Case presentation

A 5-month-old female was transferred from an outside facility for dermatologic evaluation of a diffuse pruritic and scaling rash that began at 2 months with asymptomatic dryness and erythema on the chest. By 3 months, the rash became pruritic and progressed to her face, scalp, and extremities, resulting in gradual hair shedding. Her birth history was noncontributory; no collodion membrane was present. At 5 months, the rash intensified until she developed poor oral intake and lethargy, resulting in emergency evaluation and admission for hypernatremic dehydration.

Vital signs on initial admission demonstrated hypothermia and hypotension, while bloodwork revealed hypernatremia, hypoalbuminemia, leukocytosis, and elevated transaminases. Due to her severe clinical course, she was evaluated by infectious diseases, gastroenterology, and endocrinology—all with negative respective workups. Nutritional deficiencies were considered, yet zinc, alkaline phosphatase, and total protein levels were all within normal limits. Similarly, immunologic workup yielded normal complete blood counts, immunoglobulins, and complement levels without peripheral eosinophilia. Aside from lethargy-related decreased oral intake, there were no gastroenterology symptoms. Given her altered mentation, she received empiric antibiotics and underwent lumbar puncture, which was unremarkable. The days following, she received multiple albumin transfusions and intravenous fluids; numerous attempts were made at oral feeds. Despite these efforts, hypernatremia and hypothermia persisted, requiring a radiant warmer to maintain her temperature.

**Fig 1:**
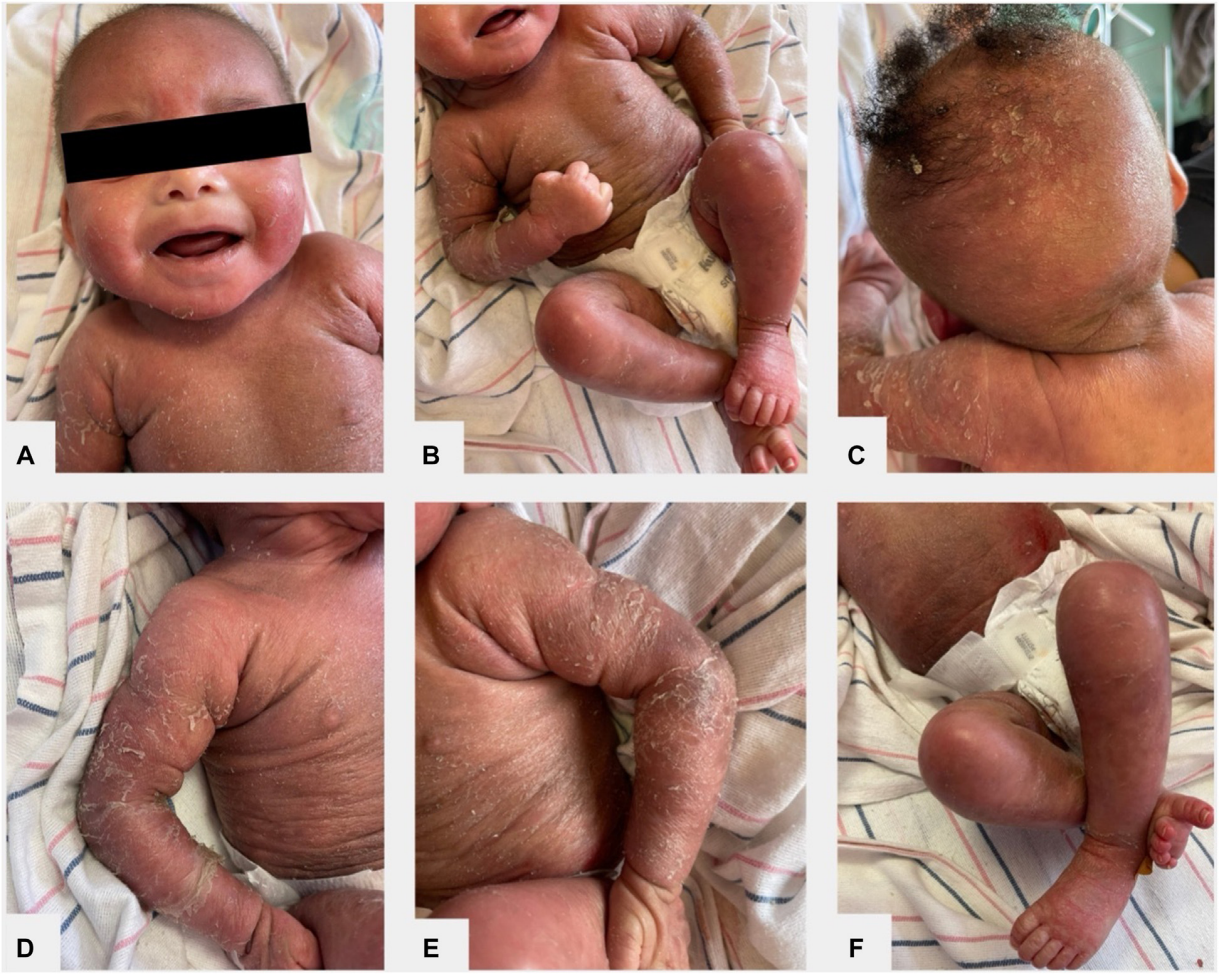
Diffuse erythematous plaques with scale and patchy erosions to the face (**A**), trunk (**B**), neck (**B**), scalp (**C**), and extremities (**D-F**).

**Fig 2:**
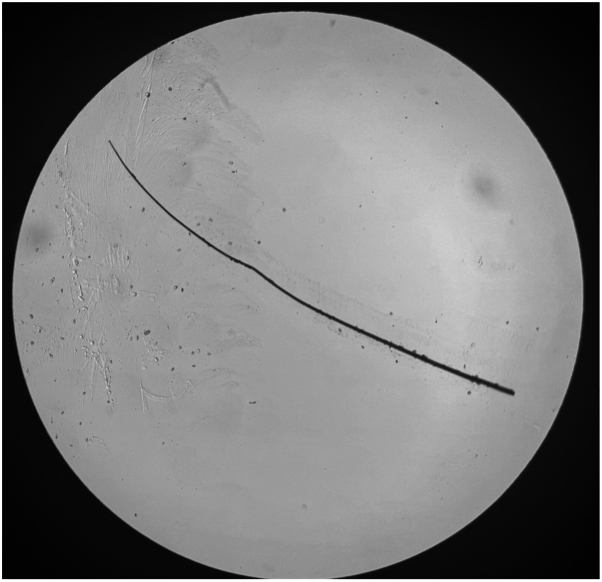
Normal hair mount findings obtained from the lateral brow without “bamboo hair” deformity.

After 13 admission days, she was transferred to our facility for inpatient dermatology services and admitted to pediatric intensive care with a sodium level of 169 mmol/L. Per literature on transepidermal water loss-induced hypernatremic dehydration, she was placed in a tent humidifier with 60% humidity. Her skin demonstrated diffuse, weeping, scaly erythematous plaques and patchy erosions to the scalp, face, neck, trunk, and extremities, with fissuring of the bilateral axillae (Fig 1). The rash spared the mucosa, flexural surfaces, palms, and soles. The hair of the scalp was diffusely thinned, but eyelashes and eyebrows were fully intact and normal length; trichoscopy showed no hair shaft abnormalities. The differential diagnosis at this time included Netherton syndrome (NS) versus severe AD; however, the normal brow hairs and eyelashes favored against NS. A punch biopsy was obtained, revealing psoriasiform hyperplasia of the epidermis without upper pallor, moderate spongiosis, normal granular layer, moderate dermal lymphocytic infiltrate, and focal, but noncompact, parakeratosis. A brow hair mount revealed normal hair shafts (Fig 2).

Clinicopathologic correlation best supported a diagnosis of severe AD. At this time, the patient was 5 months and 2 weeks of age. Given the severity of her case, the decision was made to administer an initial dose of dupilumab 200 mg subcutaneously, with subsequent injections every 4 weeks. In the next 4 days, the patient’s condition stabilized—her thermoregulation returned, sodium levels normalized at 142 mmol/L, and her skin integrity improved. Her parents were advised that her skin would improve with each injection and educated on aggressive moisturization with emollients and low-potency topical steroid ointments.

**Fig 3:**
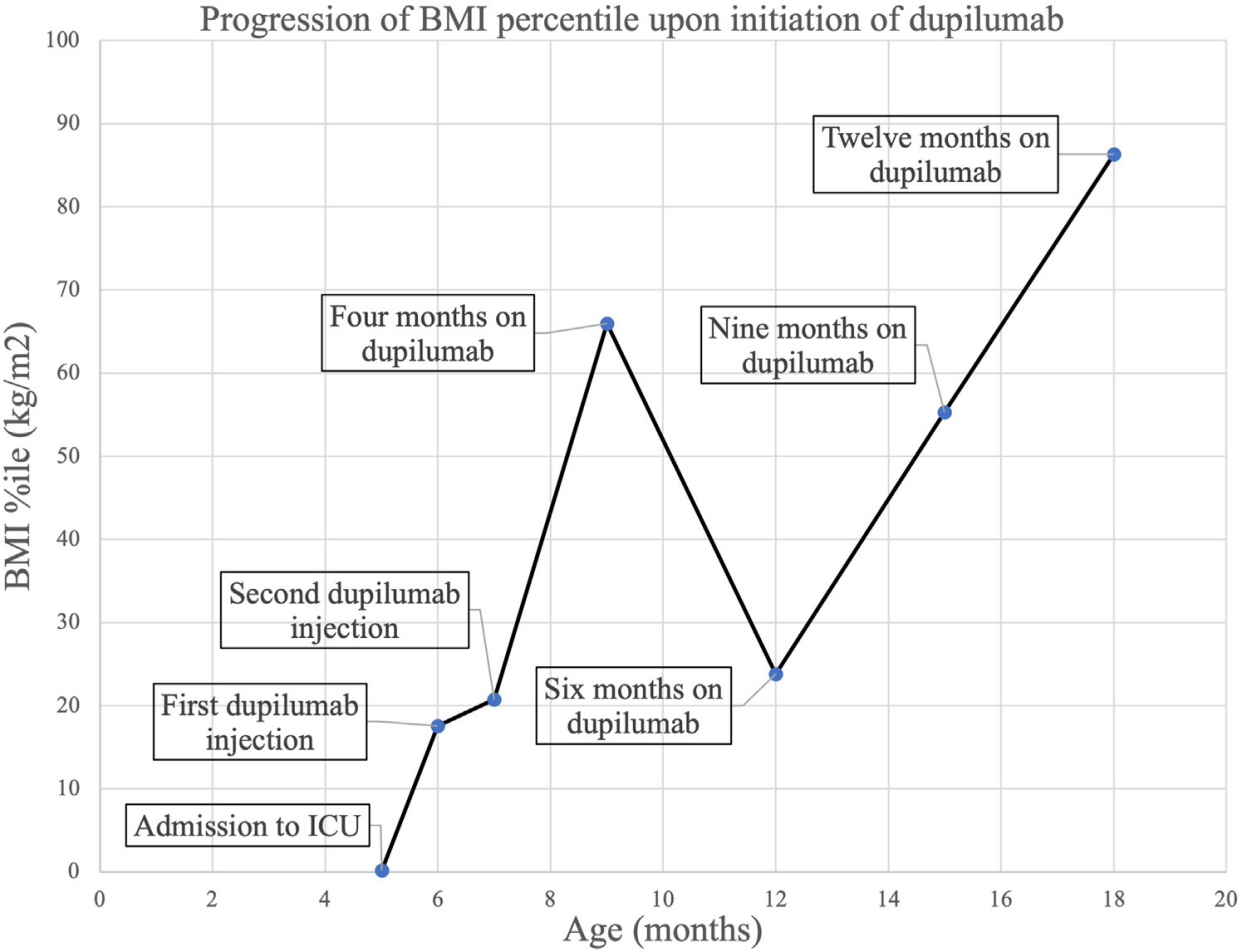
Progression of the patient’s BMI from her initial admission resulting in significant growth with dupilumab treatment. *BMI*, Body mass index; *ICU*, intensive care unit.

At her follow-up visit 1 month later, she received a second dose of dupilumab. During subsequent follow-up appointments, her skin barrier continued to improve. She continues to tolerate the dupilumab injections 1 year later, and her body mass index has advanced from her initial weigh-in at the 0.22nd percentile to most recently the 86th percentile (Fig 3). Her height and weight growth curve percentiles are similarly impressive. Her scalp hair returned in its entirety and does not exhibit breakage past a certain length.

## Discussion

While AD is typically not life-threatening, it can cause serious complications in pediatric cases, such as dehydration, electrolyte abnormalities, protein loss, temperature instability, infection, and growth retardation.^2^, ^3^, ^4^ Severe disease further impacts the psychological well-being of the patient and their caregivers. In this unique case, the patient’s FTT, hypothermia, and unusually extensive hypernatremic dehydration are direct symptoms of epidermal barrier dysfunction and transepidermal water loss caused by severe AD. The extent of her rash, scalp hair loss, and scale-like secondary features complicated the diagnosis.

Dupilumab has been trialed in a case series of 4 pediatric patients with confirmed genetic abnormalities of NS, all with hair loss involving the scalp, brows, and lashes. Despite cutaneous symptom improvement, none exhibited full hair regrowth; 1 with mild regrowth still retained “bamboo hair” deformities.^5^ Our patient’s eyebrow hair mount is still normal after 1 year on dupilumab, and her scalp hair continues to grow without breakage, supporting her hair loss as sequelae of severe AD.

Dupilumab was first approved for adults with moderate-to-severe AD in 2017 and for children as young as 6 months of age in 2022.^6^ However, the onset of AD often occurs earlier, between 3 and 6 months of age.^1^ Although our patient was short of the Food and Drug Administration-approved age, the severity of her symptoms compelled intervention, and she responded well to dupilumab without side effects. Previously, dupilumab has successfully treated a 5-month-old male with severe AD and poor weight gain.^7^ This twice-demonstrated therapeutic benefit substantiates its consideration for severe AD in patients younger than 6 months old.

## Conclusion

This case highlights the importance of mitigating severe AD’s impact on growth and development. Our patient’s hypernatremic dehydration and FTT correlated with her compromised skin barrier and resolved upon initiation and sustained dupilumab treatment. This case raises awareness of the severe sequelae of AD, promoting expert dermatologic evaluation, prompt recognition, tailored work-up, and effective treatment. In conclusion, dupilumab can be an effective management tool, but further studies are necessary to evaluate its use in infants under 6 months of age.

## Conflicts of interest

None disclosed.
